# Alleviating neurodegeneration in *Drosophila* models of PolyQ diseases

**DOI:** 10.1186/2053-8871-1-9

**Published:** 2014-07-04

**Authors:** Zhe Long, Beisha Tang, Hong Jiang

**Affiliations:** Department of Neurology, Xiangya Hospital, Central South University, 87 Xiangya road, Changsha, 410008 Hunan China; Key Laboratory of Hunan Province in Neurodegenerative Disorders, Central South University, 87 Xiangya road, Changsha, 410008 Hunan China; State Key Laboratory of Medical Genetics, Central South University, 110 Xiangyaroad, Changsha, 410078 Hunan China

**Keywords:** PolyQ diseases, *Drosophila melanogaster*, Therapeutic strategy

## Abstract

Polyglutamine (polyQ) diseases are a group of neurodegenerative conditions, induced from CAG trinucleotide repeat expansion within causative gene respectively. Generation of toxic proteins, containing polyQ-expanded tract, is the key process to cause neurodegeneration. Till now, although polyQ diseases remain uncurable, numerous therapeutic strategies with great potential have been examined and have been proven to be effective against polyQ diseases, including diverse small biological molecules and many pharmacological compounds mainly through prevention on formation of aggregates and inclusions, acceleration on degradation of toxic proteins and regulation of cellular function. We review promising therapeutic strategies by using *Drosophila* models of polyQ diseases including HD, SCA1, SCA3 and SBMA.

## Introduction

Among the unstable repeat expansion disorders, the dominantly inherited polyQ diseases caused by CAG repeat expansions within responsible genes are the most common group [1]. The first discovered polyQ disorder was Spinal bulbar muscular atrophy (SBMA), also known as Kennedy’s disease which is X-linked, in 1991, a dynamic repeat mutation in the androgen receptor (AR) gene [2]. Since then at least eight additional polyQ diseases have been identified. To date, this group includes the distinct Spinocerebellar Ataxias (SCA1, SCA2, SCA3/Machado–Joseph disease, SCA6, SCA7 and SCA17) [3–5], dentatorubral-pallidoluysian atrophy (DRPLA), Huntington disease (HD), and most recently, Huntington disease-like 2 (HDL2) [6]. Generally, compared with other neurodegenerative diseases, polyQ diseases have low prevalence. 1 ~ 3/100 000 Europeans have autosomal dominant cerebellar ataxias (ADCAs) [7], such as SCA1, SCA2, SCA3/MJD, SCA6, SCA7 and SCA17, while 1.6/100 000 individuals have SBMA [8] and 5 ~ 7/100 000 white people suffered HD [9].

In all polyQ disorders, the abnormally translated polyglutamine domain in the corresponding disease protein would lead to dysfunction and pathogenic effects, when the CAG repeat expansion in respective causative genes surpasses a pathological threshold. The involvement of the polyQ-expanded domain in pathogenic mechanisms has been illustrated in various ways. For toxic effects, several intracellular molecular mechanisms have been illustrated, including formation of aggregates [10–14], dysregulation of cellular protein homeostasis [15–18], alternations in transcription [19–24], impairment of axonal transport [25, 26], mitochondrial dysfunction [27–29] and harassment of intracellular Ca^2+^ homeostasis [30–34]. PolyQ disorders share numerous common traits including progressive neurodegeneration in specific neuronal populations, formation of protein aggregates and the negative correlation between the number of CAG repeats and the age of onset which means the greater number of such repeats leads to the earlier onset of the diseases [35–38]. Despite these common aspects, however, there are respective aspects to polyQ diseases as well. Each polyQ disease is a distinctive disorder with characteristic symptomatic profiles and different neurodegeneration occurring in particular brain regions. Although the expanded proteins are prevalently expressed throughout the CNS, there are two remarkable exceptions: SCA6 and Kennedy’s disease. The CACNA1A calcium channel subunit in SCA6 is primarily expressed in affected cerebellar Purkinje cells, while the AR in Kennedy’s disease is principally expressed in vulnerable motor neurons. Additionally, the various properties of each of PolyQ disease proteins differ in subcellular localization, structure, activity and biological function, suggesting that the specific details of pathogenic effects may be unique to each disease [2].

## Review

### Advantages of *drosophila*as genetic models for polyQ disease

Application of animal models is a powerful approach to address some of the outstanding questions underlying polyQ disease. Although modelling neurological diseases in rodent has been of significant impact, using *drosophila melanogaster* model offers many advantages for pathogenic mechanism and therapeutic compounds. Due to the shorter life span, rapid reproductive cycle, accessibility of several techniques and tools to modulate gene expression, and comparatively well-known anatomy and phenotypes, fruit fly prefer faster polyQ modelling [39, 40]. In addition, using GAL4/UAS system [41], we can manipulate the expression level of transgene and expression in specific cell types [42]. *Drosophila* and human genomes are characterized a high degree of conservation in fundamental biological pathways [43]. With the few chromosome number, convenient genetic operation, simple inherited background, *drosophila* model has great value in functional analysis of human disease genes. Moreover, high-throughput pharmalogical screens are also possible due to the minimal barrier in central nervous system, although flies have a complex nervous system and brain [44]. Up till now, several *Drosophila* models of polyQ diseases have been developed, including SCA1, SCA2, SCA3/MJD, SCA7, SCA17, DRPLA, HD and SMBA. Moreover, several human pathological features, including formation of inclusions, neural degeneration, motor dysfunction and premature lethality, have successfully been recapitulated in *Drosophila* models of polyQ [45–49].

### Therapeutic strategies for polyQ diseases

According to pathogenic hypothesis mentioned above, therapeutic strategies could target on the general toxic mechanism triggered by expanded polyQ. Generally, therapeutic strategies classified into targeting prevention on formation of aggregates and inclusions, targeting acceleration on degradation of toxic proteins and regulation of cellular function (Table 1).

**Table 1:** **Therapeutic strategies tested in** ***Drosophila*** **model of polyQ diseases**

Therapeutic strategy	Functional mechanism	Drosophila model	Therapeutic result	Reference
RNAi	Inhibiting formation of polyQ-expanded protein	SCA1, SCA3, HD	Positive	Mallik et al. [50]
Cystamine	reducing Huntingtin (Htt) aggregation	HD	Positive	Apostol et al. [51]
				Agrawal et al. [52]
				Bortvedt et al. [53]
Methylene blue (MB)	Inhibiting Htt aggregation	HD	Positive	Sontag et al [54]
18b-glycyrrhetinic acid	Inhibiting Htt aggregation	HD	Positive	Schulte et al. [55]
Camptothecins	Inhibiting Htt aggregation	HD	Positive	Schulte et al. [55]
OH-camptothecin	Inhibiting Htt aggregation	HD	Positive	Schulte et al. [55]
Carbenoxolone	Inhibiting Htt aggregation	HD	Positive	Schulte et al. [55]
Polyglutamine binding peptide 1 (QBP1)	Inhibiting polyQ aggregation	SCA3/MJD	Positive	Nagai et al. [56]
P42	Inhibiting polyQ-hHtt aggregation	HD	Positive	Arribat et al. [57]
Hsp70	Reversing toxic structure of polyQ-expanded proteins	SCA3/MJD, SBMA	Positive	Warrick et al [58]
				Wang et al [59]
Hsp40	Reversing toxic structure of polyQ-expanded proteins	HD	Positive	Kazemi et al [49]
				Kuo et al. [60]
Hsp110	Reversing toxic structure of polyQ-expanded proteins	HD	Positive	Kuo et al. [60]
Hsp22	Reversing toxic structure of polyQ-expanded proteins	SCA3/MJD	Positive	Li et al. [61]
Rapamycin	Accelerating toxic proteins degradation	HD	Positive but various side-effects on human	Ravikumar et al. [62]
				Morrisett et al. [63]
				Letavernier et al. [64]
				Kuypers et al. [65]
				Maroto et al. [66]
Lithium chloride (LiCl)	Accelerating toxic proteins degradation	SCA3/MJD	Positive	Jia et al. [67]
Normal ataxin-3	Accelerating toxic proteins degradation	SCA3/MJD	Positive	Warrick et al. [68]
Hsp104	Accelerating toxic proteins degradation	SCA3/MJD	Positive	Cushman et al. [69]
Sodium butyrate	Transcriptional regulation	HD	Positive	Steffan et al. [70]
Suberoylanilide hydroxamic acid (SAHA)	Transcriptional regulation	HD	Positive	Steffan et al. [70]
Trichostatin A (TSA)	Transcriptional regulation	SCA3/MJD	Positive	Jung et al. [71]
Valproic acid (VPA)	Transcriptional regulation	SCA3/MJD	Positive	Yi et al. [72]
Myc	Transcriptional regulation	SCA3/MJD	Positive	Singh et al. [73]
Uncoupling proteins (UCPs)	Ameliorating mitochondrial dysfunctions	HD	Positive	Besson et al. [74]
Meclizine	Ameliorating mitochondrial dysfunctions	HD	Positive	Gohil et al. [75]

### Prevention on formation of aggregates and inclusions

**Figure 1:**
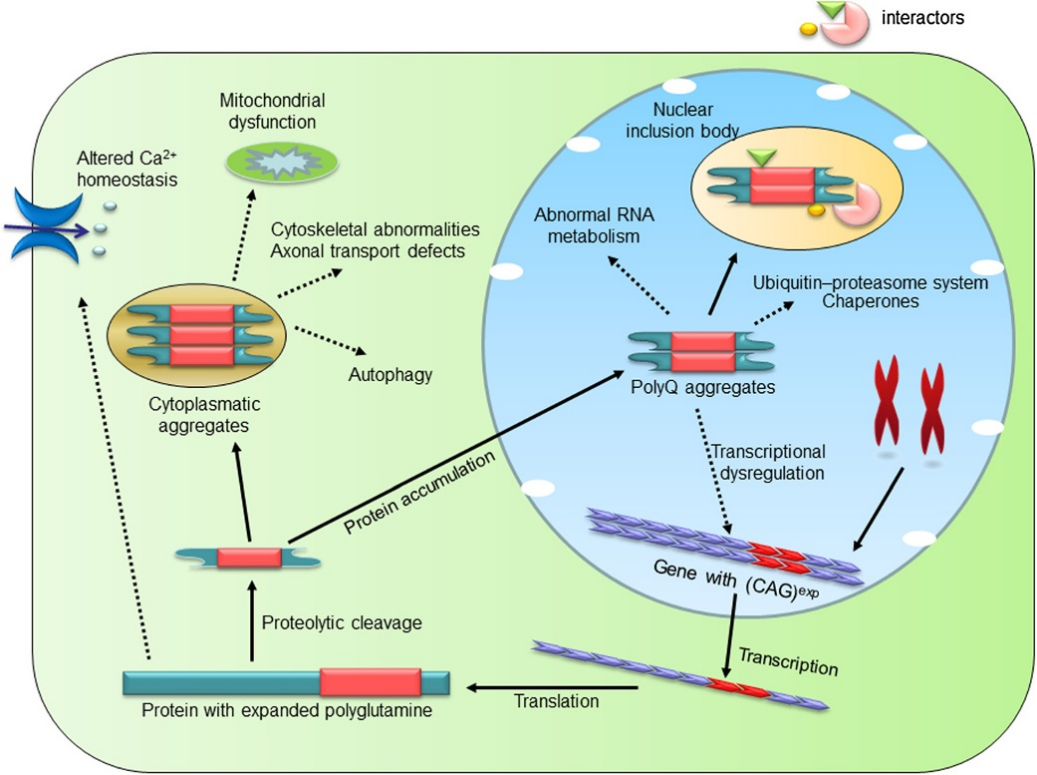
**The intra-cellular sites of toxic effects of proteins in polyQ disorders.** PolyQ aggregates induce cytotoxic effects through a range of mechanisms. To form insoluble inclusions, compiling of polyQ-expanded protein provoke quality-control mechanisms including ubiquitin–proteasome system, chaperones and autophagy. By interacting with transcriptional factors, the toxic polyQ proteins could regulate transcriptional processes. Other cellular sites of toxic effects induced by polyQ mutant proteins include Ca^2+^ channel, mitochondria, and cytoskeleton with diverse abnormalities respectively.

Insoluble aggregates present toxic effects for neurons and result in cell death and organism pathology [11]. PolyQ diseases seem to be originated by proteolytic cleavage of mutant protein containing polyQ-expanded tract to form toxic fragments [9, 10], which aberrantly fold into amyloid-like aggregates (oligomers), then assemble into nuclear and cytoplasmic deposits which are the cellular hallmarks of polyQ diseases. The neuronal protein aggregates mainly present in nucleus (SCA1, SCA2, SCA3/MJD, SCA7, SCA17 and DRPLA), cytoplasm (SCA6) or both cytoplasm and nucleus (HD and SBMA) (Figure 1) [76, 77].

#### RNA interference-based therapeutics

Mutant polyglutamine protein expression could be inhibited by RNA interference (RNAi). Inhibiting formation of mutant protein containing expanded polyQ tract, RNAi is the most straightforward approach to selectively decrease expression of the mutant allele. Xia et al. and Harper et al. have successfully testified that administration of RNAi, against *ATXN1* or *IT15*, improved the motor impairment in SCA1 and HD transgenic mice models [78, 79]. Similarly, in several *Drosophila* models of human polyQ disorders including SCA3/MJD, SCA1 and HD, Mallik et al. [50] discovered that RNAi suppress pathogenesis of polyQ diseases by down-regulating transcripts of *hsr-ω*, a dominant modifier for polyQ pathogenic mechanism found in *Drosophila* model of SCA1 [42]. Co-expressing hsrω-RNAi transgenes suppresses expanded polyQ-induced eye-specific degeneration, diminishes toxicity of mutant polyQ protein in nervous system and inhibits polyQ protein aggregation.

#### Therapeutic pharmacological compounds

A variety of chemical compounds with great potential have been proven effective for treatment of polyQ diseases by using *Drosophila* model of HD through preventing polyQ-expanded aggregation. Cystamine, methylene blue (MB), camptothecin, OH-camptothecin, 18b-glycyrrhetinic acid and carbenoxolone are promising therapeutics for HD. Cystamine, a competitive inhibitor of tissue transglutaminase (tTG), was thought to reduce Huntingtin (Htt) aggregation by interfering with tTG-mediated glutamine crosslinking [51, 52]. Bortvedt el at discovered that in adult HD flies co-treatment of feeding cystamine and expressing a transgene encoding the anti-htt intracellular antibody (intrabody) C4-scFv leads to alleviation of photoreceptor neurodegeneration without benefit in longevity, however in larval and adult stages of *Drosophila* feeding cystamine showed opposite effect: longevity was prolonged without photoreceptor rescue [53]. MB has reported promotion of degradation androgen receptor polypeptides [80] and inhibition of mutant Htt aggregation [81]. Sontag et al. found that administrating MB to *Drosophila* of HD significantly increased the rhabdomeres number accompany with a decrease in Htt-mediated neurodegeneration. In addition, treating MB in the larval stages reduced the aggregates number by 87% and diminished the aggregates size, suggesting that MB could be promising therapeutic drug for HD through the regulation of aggregation [54]. Additionally, Schulte el at by screening the subcellular distribution of Htt labeled by mRFP and monitoring the morphology of cultured neurons via live-imaging discovered 18b-glycyrrhetinic acid, as well as camptothecins, OH-camptothecin, carbenoxolone inhibited formation of Htt aggregate, restored neurite morphology and viability in *Drosophila* HD model [55].

#### Molecular therapeutics

**Figure 2:**
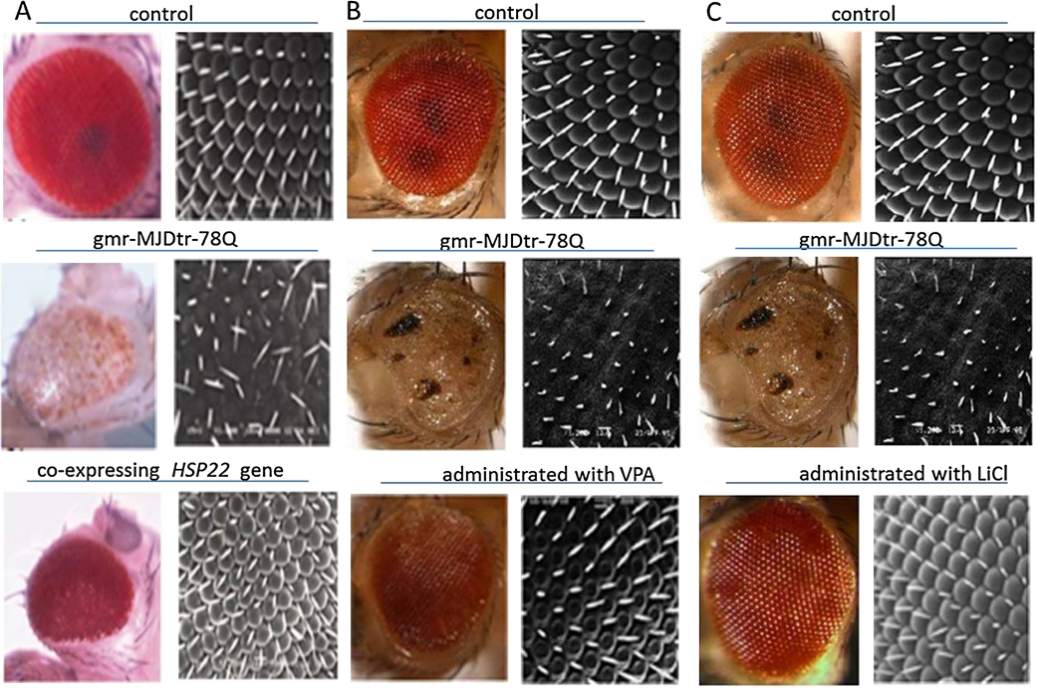
**SCA3/MJD** ***Drosophila*** **that expresses polyQ-expanded tracts in fly compound eyes.** Overexpression of Hsp22, VPA and LiCl rescues polyQ-induced eye depigmentation in *Drosophila* model of SCA3/MJD. **(A)** Hsp22, **(B)** VPA, **(C)** LiCl. Paired images of adult fly eyes are showing, dissecting microscope (**A**, ×65; **B**, ×115; **C**, ×80) and electron microscope (×1000).

Accumulation of aggregates to form inclusions recruits cellular normal proteins, such as molecular chaperones, which suggests that polyQ-expanded domain changes protein structure and activates chaperones against protein misfolding [82]. The finding that expanded polyQ tract is capable of transiting into diverse conformations is also affirmed by evidence that, in fly (SCA3/MJD, HD) and mouse (SCA1) models, overexpression of molecular chaperones represses toxicity [83, 84]. Thus, several biological molecules may thought to be potential therapeutics targeting prevention on formation of pathogenic aggregates and toxic structure of polyQ proteins, such as polyglutamine binding peptide 1 (QBP1), a peptide P42, chaperones heat shock proteins including heat shock protein 70 (Hsp70), Hsp40, Hsp110 and Hsp22. Application of QBP1 which is capable of selectively binding to the polyQ-expanded stretch suppresses compound eye degeneration, polyQ aggregates formation and rescues premature death in SCA3/MJD *Drosophila* model [56], suggesting that QBP1 is a potential therapeutic molecule on polyQ disorders. P42, a 23 amino acid-long peptide which is a part of the endogenous Htt protein, plays a protective role in preventing polyQ-hHtt aggregation, improving the impaired axonal transport by restoring the total number and motion of vesicles, ameliorating behavioral dysfunctions and against polyQ-hHtt induced toxicity in HD *Drosophila* model. However, no protective effects were found in other polyQ diseases [57]. Although the toxic conformation of polyQ proteins remains elusive, therapeutic strategy targeting on toxic structure of expanded polyglutamine proteins may be a promising approach against untreatable polyQ disease. In *Drosophila melanogaster* model of SCA3/MJD, co-expressing the human gene *HSPA1L* which encodes Hsp70, Warrick et al. discovered that Hsp70 completely rescued external eye pigmentation, partially restored retinal structure of brain, and partially restored adult viability, suggesting that molecular chaperone Hsp70 suppresses polyglutamine-induced neurodegeneration and toxicity as well as indicating that HSP70 would be a promising candidate with great potential as a treatment method [58]. Additionally, in a *Drosophila* model of SBMA, Adrienne et al. demonstrated that Hsp70 with its co-chaperone Hip which enhances Hsp70 binding to its substrates accelerated polyQ AR clearance, and identified that Hsp70 with YM-1, a synthetic co-chaperone that acts similarly to Hip, also enhanced polyQ AR degradation and suppressed toxicity of poyQ AR in *Drosophila* [59]. Similarly, Kazemi et al. by screening the fly genome for genes modulating the toxicity of polyglutamine, predicted dHDJ1, homologous to human Hsp40/HDJ1, and dTPR2, homologous to the human tetratricopeptide repeat protein 2 (TPR2) suppressed the toxicity of polyQ aggregates and verified in *Drosophila* models of polyQ diseases [49]. In addition, Y Kuo el at further demonstrated that co-expression of the HSP40 family protein DNAJ-1 and Hsp110 family protein, 70 kDa heat-shock cognate protein cb (HSC70cb), function together to suppress the cytotoxicity of mutated huntingtin in *Drosophila* HD model. Furthermore, DNAJB1, a human Hsp40, co-expressed with APG-1, a human Hsp110, in cells from HD *Drosophila* had a dramatic protective effect on polyQ-induced neural degeneration, whereas either component alone had little effect [60]. In our previous studies, using several types of SCA3/MJD *Drosophila* models, we have provided convincing proof that Hsp22 would be promising therapeutic agents with great potential against SCA3/MJD. Expression of MJDtr-Q78, a polyQ-expanded tract, showed significantly obvious SCA3/MJD phenotype including dramatic neurodegeneration, and completely faded pigmentation in adult flies with black point-like necrosis. *Drosophila* co-expressed polyQ-expanded protein together with either one or two copies of *HSP22* gene intervened by heat shock, leading to differing corresponding mRNA levels mainly depending on the induced number of *HSP22* gene copies. Findings suggested that Hsp22 showed positive influence on eye depigmentation (Figure 2), growth restriction, ability for eclosion and median lifespan [61].

### Acceleration on degradation of toxic proteins

Stimulation of cellular protein degradation mechanisms that target misfolded causative mutant proteins might be potential against polyQ diseases. Activation of autophagy and several small molecules presented positive influence on disease proteins clearance. By stimulating autophagy, rapamycin, the mTOR inhibitor, suppressed neurodegeneration in cell, *Drosophila* and mouse disease models [62, 85, 86] However, the various deleterious side-effects including dysfunction of kidney and lung, increased risk of infection and hyperlipidemia, prevent it from widely using in polyQ diseases [63–66]. In HD *Drosophila* model, lithium (Li) acting through the Wnt/Wg pathway, as a glycogen synthase kinase-3 (GSK3) β-specific inhibitor, showed protective effects against the toxicity caused by aggregates of causative polyQ proteins [87]. Similarly, since Li plays neuroprotective role in numerous models of neurodegenerative disorders and could induce autophagy for reducing the mutant protein aggregates, in our previous study, we confirmed its neuroprotective effects against protein toxicity induced by polyQ repeat expansion. As SCA3/MJD is the most common spinocerebellar ataxia in minland China [88, 89], we used SCA3/MJD *Drosophila* model, and expression of MJDtr-78Q in *Drosophila* lead to late-onset, progressive, neurodegenerative phenotypes, including faded eye pigmentation, impaired locomotor ability and reduced life spans in adult flies. A series of daily dose of lithium chloride (LiCl) were administered to SCA3/MJD Drosophila model prior to cross-breeding. We affirmed that long-term use of LiCl at specific doses notably inhibited eye depigmentation (Figure 2), alleviated locomotor disability, and prolonged the median lifespan [67]. Additionally, it is well acknowledged that the pathogenesis of SCA3/MJD is involved with pathogenic ataxin-3 induced by expanded polyglutamine repeat in *ATXN3*. Interestingly, normal human ataxin-3 is thought to be a neuroprotective protein that acts as a polyubiquitin binding protein with ubiquitin protease activity [90–93]. In *Drosophila*, normal ataxin-3 rescues neurodegeneration from expanded polyQ proteins, highlighting its potential therapeutic role for polyQ diseases [68]. Another small molecule, Hsp104,a protein disaggregase, can rapidly resolubilize denatured protein aggregates and restore function of proteins [94, 95]. Recently, in SCA3/MJD *Drosophila* model, proof has been provided that Hsp104 suppressed toxicity of pathogenic protein, mitigating disease progression [69].

### Regulation of cellular function

#### Transcriptional regulation

Toxic polyglutamine proteins inclined to deposit in the nucleus, indicating that these compiling proteins might interact with transcriptional factors or cofactors and lead to alterations of transcription. Interactions of these acumulating proteins with specific transcriptional factors or cofactors may disturb gene expression, and thus initiate neurodegeneration. Therapeutic strategy target on this has been testified neuroprotective effects against polyQ diseases. Histone acetylation and deacetylation is a post-translational modification of proteins, which regulates gene transcription by changing the compactness of nucleosome polymers. An imbalance in histone acetylation may be a key process causing transcriptional dysregulation involved in polyQ diseases. Several histone deacetylase (HDAC) inhibitors have been proven increase gene expression in diverse disease models, such as suberoylanilide hydroxamic acid (SAHA), sodium butyrate, and phenylbutyrate [50, 96–100]. In *Drosophila* models of HD, HDAC inhibitors, including sodium butyrate and SAHA, prevent developing progressive neuronal degeneration caused by mutant polyglutamine repeat expansion, and reduce lethality. These findings present therapeutics for polyQ diseases, even after the onset of symptoms, treatment with HDAC inhibitors are still capable of slowing or arresting the progressive neurodegeneration [70]. Adenosine 3′, 5′-monophosphate (cAMP) response element–binding protein (CREB)–binding protein (CBP) [101, 102], a histone acetyltransferase (HAT) found in polyQ inclusions, its decreased activity contributes to polyQ disease. In SCA3/MJD *Drosophila* model, trichostatin A (TSA) protected against the increased rate of repeat instability, by compensating for decreased *Drosophila* CBP and/or HAT activity [71]. Additionally, we utilized HDAC inhibitor valproic acid (VPA) [72], a potential therapeutic agent, in *Drosophila* SCA3/MJD model. Deleterious phenotypes, including faded eye pigmentation, decreased climbing ability and shortened average life span, were similar to characteristics of SCA3/MJD patients. To examine the therapeutic effects of VPA in vivo, daily doses of VPA at various levels were administered to SCA3/MJD *Drosophila* before cross-breeding. We suggested that chronic treatment with VPA at optimum dose partly arrested eye depigmentation (Figure 2), ameliorated climbing disability, and prolonged the average lifespan of SCA3/MJD transgenic *Drosophila*.

Additionally, present researches direct to detect genetic modifiers that rescue polyQ degeneration, thus provide a novel target for testing therapeutic strategies. Recenly, Yamanaka et al applied RNAi screening in mouse neuro2a cells to identify modifiers for mutant huntingtin aggregates, and identified 111 shRNAs including 63 shRNAs suppressing huntingtin aggregation and 48 shRNAs performing an opposite effect [103]. We look forward to further study on testing neuroprotective effects of these 63 shRNAs for HD in vivo. Another study on genetic modifier in SCA3/MJD model of *Drosophila* confirmed that up-regulation of dMyc, a homologue of human cMyc proto-oncogene, could restore morphology and functional vision of eyes and suppress lethality by increased cellular level of CBP, which subsequently improve the status of histone acetylation and finally inhibit the compiling of polyQ aggregates [73].

#### Amelioration of mitochondrial dysfunctions

Compared with polyQ disease proteins, expanded CAG mutant transcripts are different polyQ pathogenic species to induce neurodegeneration [104]. Nucleolar stress is a p53-mediated pathway through which the nucleolus communicates with mitochondria to induce apoptosis by down-regulation of ribosomal RNA (rRNA) expression [105]. The nucleolar stress is activated by the expanded CAG mutant transcripts interacting directly with nucleolin (NCL), an essential protein for rRNA transcription, resulting in down-regulation of NCL binding to *upstream control element (UCE)* of the *rRNA promoter* [106], subsequently, *UCE* DNA CpG hypermethylation and the down-regulation of rRNA transcription [104]. Consequently, apoptosis is provoked by p53 protein accumulating in the mitochondria, which causes cytochrome c release and caspase activation [106]. Additionally, evidence that mitochondrial dysfunction is correlated with HD pathogenesis has been presented, by identified N-terminal expanded htt on neuronal mitochondrial membranes through electron microscopy [27]. According to these, numerous biological molecules and pharmacological compounds have shown efficacy in treatment for polyQ diseases, particularly for HD. Mitochondrial uncoupling proteins (UCPs), anion-carrier proteins located in the inner membrane of mitochondrial, modulate ATP and reactive oxygen species in mitochondria. Although UCPs failed to prevent the HD toxicity in neurons, by using genetic approaches in *Drosophila*, up-regulation of UCPs alleviated the HD locomotor behavior and early death of *Drosophila* when mHtt was selectively expressed in glia [74]. Additionally, inhibition of transglutaminase 2 (TG2), a transcriptional co-repressor, not only corrects transcriptional dysregulation in HD but also normalizes expression of mitochondrial genes, and protect neurons from excitotoxicity [107]. Meclizine, a clinically used drug that has correlated with mitochondrial respiration suppression, in *Drosophila melanogaster* models of HD, played a neuroprotective role against neuronal dystrophy and cell death, implicating that meclizine, capable of crossing the blood–brain barrier, may hold therapeutic potential against HD [75].

## Conclusion

To date, targeting the pathogenic mechanisms, the fact that various therapeutic strategies have shown protective efficacy in *Drosophila* models of polyQ diseases gives new insight to researchers and clinical doctors as well as presents hope for patients of these diseases. Unfortunately, only a few of these therapeutic strategies have been testing in clinical trial, and the clinical efficacy still illusive. Anyhow, as *Drosophila melanogaster* presents various advantages and convenience, we can anticipate that in the future more therapeutic targets will be discovered by further understanding the pathogenic mechanisms on polyQ diseases, thus increasingly biological molecules and pharmacological compounds for polyQ diseases might be tested by employing *Drosophila* models, and these models of polyQ diseases could provide diverse options and clues for clinical application.

### Ethical approval

The study was approved by the Expert Committee of Xiangya Hospital of Central South University in China (equivalent to an Institutional Review Board).

## Abbreviations

polyQ: Polyglutamine
SBMA: Spinal bulbar muscular atrophy
AR: Androgen receptor
SCA: Spinocerebellar ataxias
DRPLA: Dentatorubral-pallidoluysian atrophy
HD: Huntington disease
HDL2: Huntington disease-like 2
ADCAs: Autosomal dominant cerebellar ataxias
RNAi: RNA interference
MB: Methylene blue
tTG: Tissue transglutaminase
Htt: Huntingtin
intrabody: Intracellular antibody
QBP1: Polyglutamine binding peptide 1
Hsp: Heat shock protein
TPR2: Tetratricopeptide repeat protein 2
HSC70cb: 70 kDa heat-shock cognate protein cb
Li: Lithium
GSK3: Glycogen synthase kinase-3
LiCl: Lithium chloride
HDAC: Histone deacetylase
SAHA: Suberoylanilide hydroxamic acid
TSA: Trichostatin A
CBP: Adenosine 3′, 5′-monophosphate (cAMP) response element–binding protein (CREB)–binding protein
HAT: Histone acetyltransferase
VPA: Valproic acid
rRNA: Ribosomal RNA
NCL: Nucleolin
UCE: Upstream control element
UCPs: Uncoupling proteins
TG2: Transglutaminase 2.
